# Supplementary figures and images for: Cystatin C: immunoregulation role in macrophages infected with Porphyromonas gingivalis
Source: PeerJ. 2024 Apr 30;12:e17252. doi: 10.7717/peerj.17252 (PMC11067906; doi:10.7717/peerj.17252)

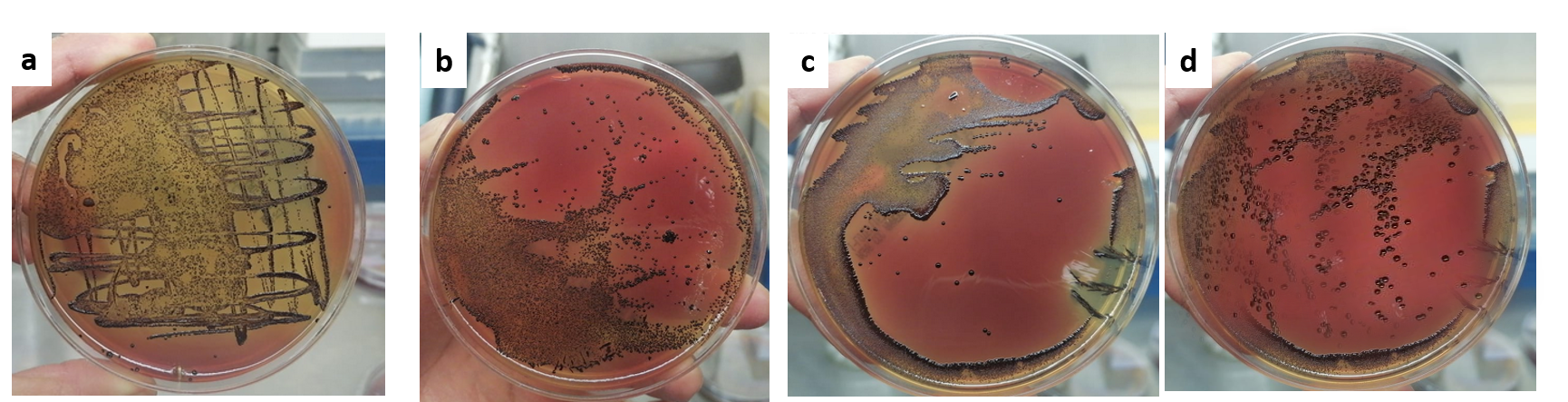

Supplement: Supplemental Information 2 — a) Lysates of macrophages infected with P. gingivalis. Lysates of macrophages infected with P. gingivalis and stimulated with Cystatin C, for b)24 h, c)48 h, d)96h [file peerj-12-17252-s002.png]
